# COSUTI: a protocol for the development of a core outcome set (COS) for interventions for the treatment of uncomplicated urinary tract infection (UTI) in adults

**DOI:** 10.1186/s13063-019-3194-x

**Published:** 2019-02-07

**Authors:** Sinead Duane, Akke Vellinga, Andrew W. Murphy, Martin Cormican, Andrew Smyth, Patricia Healy, Michael Moore, Paul Little, Declan Devane

**Affiliations:** 1 HRB Trials Methodology Research Network, College of Medicine, Nursing & Health Sciences, National University of Ireland, Galway, Ireland; 2 Discipline of General Practice, School of Medicine, National University of Ireland, Galway, Ireland; 3 Discipline of Bacteriology, School of Medicine, National University of Ireland, Galway, Ireland; 4 Discipline of General Practice, HRB Primary Care Clinical Trial Network Ireland, College of Medicine, Nursing and Health Sciences, National University of Ireland, Galway, Ireland; 5 HRB Clinical Research Facility Galway, National University of Ireland, Galway, Ireland; 6 School of Nursing and Midwifery, National University of Ireland, Galway, Ireland; 7 Primary Care and Population Sciences, Primary Care and Population Sciences, University of Southampton Faculty of Medicine, Southampton, UK

**Keywords:** Core outcome set, Urinary tract infections, Cystitis, Delphi survey, Systematic review

## Abstract

**Background:**

Urinary tract infections (UTIs) are the second most common infection presenting in the community. Clinical guidelines and decision aids assist health practitioners to treat a UTI; however, treatment practices vary due to patient needs and context of presentation. Numerous trials have evaluated the effectiveness of treatment interventions for UTI; however, it is difficult to compare the results between trials due to inconsistencies between reported outcomes. Poor choice of outcome measures can lead to impairment of evidence synthesis due to the inability to compare outcomes between trials with similar aims. Transparency in selecting and reporting outcomes can be mitigated through the development of an agreed minimum set of outcomes that should be reported in clinical trials, referred to as a core outcome set (COS). This paper presents the protocol for the development of a COS for interventions in the treatment of uncomplicated UTI in adults.

**Methods:**

This COS development consists of three phases. Phase 1 is a systematic review, which aims to identify the core outcomes that have been reported in trials and systematic reviews of interventions treating uncomplicated UTI in adults. Phase 2 consists of a three-round online Delphi survey with stakeholders in the area of treatment interventions for UTI. The aim of this online Delphi survey is to achieve consensus on the importance of the outcomes emerging from Phase 1 of this research. Phase 3 is a consensus meeting to finalise the COS that should be reported in trials evaluating the effectiveness of interventions for the treatment of UTI.

**Discussion:**

It is hoped that the development of a COS for interventions for the treatment of uncomplicated UTI in adults will be adopted as a minimum set of outcomes that should be reported and measured within this context. If the findings from clinical trials related to treatment interventions for UTI are to impact on policy and practice, it is important that the findings from different treatment interventions are comparable across trials.

**Electronic supplementary material:**

The online version of this article (10.1186/s13063-019-3194-x) contains supplementary material, which is available to authorized users.

## Background

Urinary tract infections (UTIs), sometimes referred to as cystitis or lower UTI, are the second most common infection presenting in primary care [1]. In the United States, it is estimated that UTIs result in 8.6 million healthcare visits and cost the healthcare system 1.6 million dollars per annum [2]. UTIs are more common in females, with the incidence thought to be 50 times higher than their male counterparts [3]. More than 30% of women will experience a UTI in their lifetime [3, 4]. The diagnosis of a UTI requires consideration of symptomatic presentation as well as laboratory testing [5]. Common symptoms include urgency, frequency, and dysuria [1]. The most common cause of an uncomplicated UTI is *Escherichia coli* [6]. Treatment of an uncomplicated UTI is often empirical, meaning that treatment decisions are based on symptoms which are unconfirmed by microbiological tests [7]. Debate continues as to the most appropriate treatment for a UTI as practice can vary in relation to interpretation of symptoms and treatments [8].

Clinical trials are robust designs used to evaluate the effectiveness of healthcare interventions. However, the impact of their results on policy and practice may be limited by a lack of consistency in outcomes measured and reported across trials. This heterogeneity in outcomes makes it difficult to synthesise findings across trials and limits the ability of evidence to inform healthcare decisions [9]. In addition, the choice of outcomes may not reflect the views of all groups with a stakeholding interest.

Transparency in selecting and reporting outcomes can be mitigated through the development of an agreed minimum set of outcomes that should be reported in clinical trials. This is known as a core outcome set (COS) [10]. Reporting a minimum, standard set of core outcomes within and across trials of a similar condition, such as trials of UTI treatment interventions, can facilitate data synthesis and reduce reporting biases [11]. The development and use of COS is promoted by the Core Outcome Measures in Effectiveness Trials (COMET) initiative. Since its establishment in 2011, the COMET repository has over 1000 references to planned, ongoing, or completed COS work across a variety of health conditions [12].

### Aims and objectives

This paper presents the protocol for the development of a COS for interventions in the treatment of uncomplicated UTI in adults.

The objectives are: 1) to conduct a systematic review to identify a comprehensive list of outcomes reported in trials examining the effectiveness of interventions for the treatments for uncomplicated UTI in adults; and 2) to develop consensus on a COS for evaluation of interventions for the treatment of uncomplicated UTI through a modified Delphi survey and consensus group meeting.

## Methods

This protocol has been developed using recommendations outlined in the Core Outcome Set Handbook [10], Core Outcome Set reporting guidelines [13], and guidance from a stakeholder advisory group made up of healthcare professionals (general practitioners, microbiologists, nephrologists, and epidemiologists), researchers in the field of UTI and trial methodology including COS development, and members of the public affected by UTI. A SPIRIT Checklist has been included as an Additional file 1.

The COS development will encompass three phases: Phase 1 is a systematic review identifying the core outcomes that have been reported in randomised trials and systematic reviews of randomised trials of interventions for the treatment of uncomplicated UTI in adults; Phase 2 is an online, three-round Delphi survey with stakeholders; and Phase 3 is a consensus meeting.

### Phase 1: systematic review

#### Types of studies

Studies are all randomised trials and systematic reviews of randomised trials (with and without meta-analyses) comparing the effectiveness of any interventions for the treatment of uncomplicated UTI in adults.

#### Types of interventions

All trials investigate the effectiveness of treatment interventions for uncomplicated adult UTI. For the purpose of this research, uncomplicated UTI is defined as the acute onset of dysuria, frequency, or urgency in healthy male and non-pregnant woman without known functional or anatomical abnormalities of the urinary tract [14]. Papers which refer to uncomplicated cystitis are also included. Papers primarily reporting the treatment of recurrent UTIs have been excluded from this study. Recurrent UTIs are defined widely as two infections in 6 months or three or more in 1 year [15]; these types of patients are often included in studies concerned with prophylaxis, risk factors, and self-initiated management, which are beyond the scope of this study [16]. Studies that are primarily investigating treatments for the signs and symptoms of pyelonephritis are also excluded.

Treatment interventions are defined as “anything that aims to make a change to someone’s health. For example, providing a counselling service, giving a drug, or giving people information and training are all described as interventions” [17]. Reflecting this definition within the proposed COS, the term ‘treatment interventions’ has been kept broad and can include pharmacological, non-pharmacological, complex, and behavioural treatment interventions.

#### Types of participants

Participants are otherwise healthy adults (over 18 years old), male and non-pregnant female, who received treatment for an uncomplicated UTI. Pregnant women have been excluded as the treatment regime for a UTI in pregnancy may be different as highlighted by NICE [18] and SIGN [19] guidelines. Studies will be restricted to adults only and, where papers include adult and children, only outcomes relevant to adults will be selected.

#### Search methods for identification of studies

This systematic review will review the literature over the last 10 years (2007–2017). Citation databases that will be searched are the Cochrane Database of Systematic Reviews (including CENTRAL, CDSR and DARES), PubMed, and Embase. The PRISMA [20] and COMET [10] guidelines will be used to report the conduct and findings of this review. A combination of search terms will be used for condition, study design, and interventions. Searches were unrestricted to language, but only English language papers will be screened. The full search strategy is available in Additional file 2.

#### Assessment for eligibility

Titles and abstracts will be screened independently by at least two reviewers. Covidence, a web-based citation screening tool, will be used to manage the screening process. The full text of studies deemed potentially eligible will be obtained and screened independently for eligibility by two reviewers. Where there is uncertainty, these papers will be assessed by an additional reviewer. If there is a disagreement, these papers will be discussed with the advisory group.

#### Data extraction

Outcomes will be extracted verbatim. For papers judged eligible for inclusion, the following data will be extracted to a purposefully designed database within Microsoft Excel:

- Author details, year, and journal title
- Interventions under investigation
- All intervention outcomes reported within the trial (including definitions, tools for measurement, and time points)

#### Data analysis and presentation

Extracted outcomes will be grouped into outcome domains by one reviewer, checked by a second reviewer, and approved by the COS advisory group. Outcome domains will be a broad term or phrase that will be used to categorise outcomes that are deemed similar; for example, all outcomes relating to ‘time to cure’ will be categorised under the domain name ‘time to cure’.

### Phase 2: Delphi survey

The Delphi survey is a consensus building methodology. It involves a panel of stakeholders anonymously participating in sequential questionnaires (survey rounds) rating the importance of the reported outcomes. This survey will be administered online, which means the participants do not interact with one another thus removing group thinking.

The aim of the online Delphi survey is to offer stakeholders the opportunity to rate the importance of outcomes identified from the systematic review process (Phase 1) for inclusion in the final COS and to identify additional outcomes of importance that were not captured within the review process.

#### Types of participants/stakeholder involvement

Stakeholders who have expertise in UTI treatment interventions (for example, delivering, developing, implementing, evaluating, or experiencing interventions) will be invited to participate in the Delphi panel. Invitations to participate in the online Delphi survey will be made to three stakeholder groups: 1) members of the public who have experience in being treated for UTI; 2) healthcare professionals who have experience treating people with UTI and policy makers; and 3) researchers with expertise related to the treatment of UTI.

#### Recruitment

A snowball sampling strategy will be used to identify experts in each of the stakeholder groups who will be sent an invitation to participate via electronic means (i.e. email or social media). A list of researcher/academic stakeholder representatives will be identified through published papers related to the treatment of UTI. Healthcare professionals will be approached through relevant national and international professional organisations. Policy makers will be identified through published policies and briefs related to healthcare and UTI. Public representatives will be identified and invited to participate through established patient advocacy groups and social media activities. This process will ensure that the list of emerging outcomes is relevant and important to all stakeholder groups.

Where appropriate, gatekeepers within relevant stakeholder organisations will be asked to distribute the COS invitation through their stakeholder mailing list on behalf of the research team. The research team will provide these organisations with an email invitation. This invitation email will explain the aims of the study, what it is about, what participants are asked to do, and why their participation is important.

The invitation email will also contain an electronic link which will allow stakeholders who are willing to participate to register for the survey and provide their consent for completing all three rounds of the online Delphi survey. Registered participants will receive an email containing a link to round 1 of the survey only after they have consented to participate. The participants will then be given 2–3 weeks to complete round 1.

We aim to recruit similar numbers of participants to each stakeholder group. While there is an absence of evidence on optimal sample size for each group participating in a Delphi survey, our sample size overall, and within groups, will be guided by the COMET handbook feasibility considerations and recommendations [10]. Therefore, we will work to recruit as many stakeholders as possible using the methods described below. We want to ensure that the public stakeholder group is adequately represented and will therefore weight in favour of this group as outlined in the coreHEM study [21] (see Round 3 below).

#### Study design: data collection and management

The three-round online Delphi survey will be administered using an electronic web-based system distributed via email. Each round will be open for 3 weeks to give the stakeholders an opportunity to complete the Delphi. Participants who have not responded to the survey will be sent periodic reminders via email and a final reminder will be sent the day before the survey is closed. Participants who do not complete a round will not be invited to the next round. As per recommendations outlined by the GRADE group, each round will use the same nine-point Likert scale to rate the importance of the outcome for inclusion in the COS. A rating of ‘limited importance’ (rating of 1–3), ‘not crucial importance’ (rating of 4–6), or ‘crucial importance’ (rating 7–9) will be used [22]. Participants will also be given the option of ‘unable to score’.

#### Round 1

In the first round of the Delphi survey, each stakeholder will be asked to provide some demographic information and then rate the importance of each outcome that emerged from Phase 1. One open-ended question will be included at the end of round 1 to give participants the opportunity to suggest outcomes they feel are important but have not been included in the survey. New outcomes that have been suggested by two or more participants will be considered for inclusion in round 2.

#### Round 2

All participants who completed round 1 will be invited to round 2. All outcomes included in round 1 will be carried forward to round 2. Any new outcomes suggested by two or more respondents in round 1 will also be included. For round 2 of the survey, the participants will receive their individual score for each outcome, the aggregated scores of their stakeholder group, as well as the other stakeholder groups from the previous round to consider when they are completing the survey. Based on this feedback, each stakeholder will be asked to rate each outcome again. In addition, participants in round 2 will be invited to consider if they are willing to attend a face-to-face consensus meeting to discuss the final set of outcomes.

#### Round 3

Participants who completed round 2 will be invited to the third and final round. Round 3 of the Delphi survey will contain the list of the outcomes that are rated as critical (rated 7–9) by at least 70% of respondents and rated as of limited importance (1–3 on Likert scale) by 15% or less of all respondents in round 2. In addition, to ensure that public prioritised outcomes are not overwhelmed by other groups, any outcome that had an average public score of 7 or more was also re-proposed for voting in round 3. Each participant will then be asked to rate each outcome for a final time using the same rating scale used in rounds 1 and 2.

### Phase 3: consensus meeting

The aim of the third and final phase of this COS development will be to finalise the COS that should be reported when evaluating interventions for the treatment of uncomplicated UTI in adults.

#### Participants

Participants will include representatives of the three stakeholder groups who completed Phase 2 of this research and who indicated they would be willing to participate. A convenience sampling strategy will be adopted to ensure that meeting participants will be composed of a mixture of representatives from each of the three stakeholder groups (researchers, practitioners/policy makers, and public). It is also desirable to include national and international participants within this research. Attendees will be sent information about the results of the Delphi survey prior to attending the consensus meeting.

#### Schedule and session management

If feasible, the 1-day consensus meeting will be scheduled to coincide with an academic conference of relevance to the treatment of uncomplicated UTIs in adults. Participants may attend in person or virtually by video conference. The chair will be independent, and the facilitator will encourage all stakeholders to have equal input during the meeting, adopting a collaborative approach to achieving consensus. Outcomes rated as 7–9, crucial, by ≥ 70% of participants and 1–3, of limited importance, by fewer than 15% of participants in round 3 will be considered to meet the definition of consensus. These outcomes will be brought forward to the consensus meeting. Outcomes that are rated 1–3 by ≥ 70% of participants and rated 7–9 by less than 15% of participants in round 3 will be excluded and not discussed at the meeting. Any outcomes that do not meet either definition will be classified as no consensus and brought forward to the meeting [10, 23].

## Discussion

To our knowledge, there is currently no COS for interventions in the treatment of UTI. It is hoped that this COS will be adopted as a minimum set of outcomes that should be reported and measured within this context. The researchers propose a rigorous approach to the development of this COS, which adheres to best practice guidance from the COMET handbook, COS reporting guidelines, and other protocols which have adopted COS methodologies for other health conditions [12]. The COS incorporates the perspectives of multiple stakeholders from research/academic, health professional/policy makers, and patient communities. It is hoped that this approach will ensure that the interests of all groups are represented in outcomes measured and reported in trials evaluating effectiveness of treatment interventions for UTI in the future.

In addition, use of the COS will assist in synthesising evidence from individual studies. To further broaden the transparency of this research, the final COS will be classified within a broader outcome taxonomy developed in 2017. The purpose of this taxonomy is to classify outcomes to increase efficiencies when searching for them in clinical trial registries [24].

## Additional files

Additional file 1: SPIRIT 2013 checklist: recommended items to address in a clinical trial protocol and related documents. (DOC 113 kb)

Additional file 2: PubMed search queries for the systematic review. (DOCX 17 kb)

## Abbreviations

COMET: Core Outcome Measures in Effectiveness Trials
COS: Core outcome set
UTI: Urinary tract infection
