# Supplementary material for: COSUTI: a protocol for the development of a core outcome set (COS) for interventions for the treatment of uncomplicated urinary tract infection (UTI) in adults
Source: Trials. 2019 Feb 7;20:106. doi: 10.1186/s13063-019-3194-x (PMC6367821; doi:10.1186/s13063-019-3194-x)
Supplement: Supplementary file 1 — SPIRIT 2013 checklist: recommended items to address in a clinical trial protocol and related documents. (DOC 113 kb) [file 13063_2019_3194_MOESM1_ESM.doc]

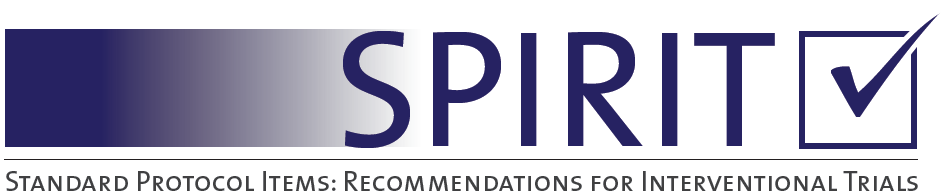


SPIRIT 2013 Checklist: Recommended items to address in a clinical trial protocol and related documents*

| Section/item | ItemNo | Description |
| --- | --- | --- |
| **Administrative information** | | |
| Title | 1 | A protocol for the development of a Core Outcome Set for interventions for the treatment of uncomplicated urinary tract infection (UTI) in adults |
| Trial registration | 2a | COS registered on the COMET Website http://www.comet-initiative.org/studies/details/950?result=true |
|  |  |  |
| Protocol version | 3 | Jan 2019 |
| Funding | 4 | Health Research Board |
| Roles and responsibilities | 5a | PP 1 |
|  | 5b | PP12 |
|  | 5c | PP12 |
|  | 5d | NA |
| Introduction |  |  |
| Background and rationale | 6a | PP 2-5 |
|  | 6b | NA |
| Objectives | 7 | PP 4 and 5 |
| Trial design | 8 | PP 5-11 |
| Methods: Participants, interventions, and outcomes | | |
| Study setting | 9 | PP 5-9 |
| Eligibility criteria | 10 | PP 6 |
| Interventions | 11a | PP 5-pg10 |
|  | 11b | NA |
|  | 11c | NA |
|  | 11d | NA |
| Outcomes | 12 | Pg5 |
| Participant timeline | 13 | Pg 5-pg10 |
| Sample size | 14 | pp 9-10 |
| Recruitment | 15 | Pg 8 |
| **Methods: Assignment of interventions (for controlled trials)** | | |
| Allocation: |  |  |
| Sequence generation | 16a | NA |
| Allocation concealment mechanism | 16b | NA |
| Implementation | 16c | Participants will opt in using an email invitation Pg8 |
| Blinding (masking) | 17a | NA |
|  | 17b | NA |
| **Methods: Data collection, management, and analysis** | | |
| Data collection methods | 18a | Pg7 |
|  | 18b | Plans to promote participant retention and complete follow-up, including list of any outcome data to be collected for participants who discontinue or deviate from intervention protocols are as stated in our ethics application (as highlighted below), this text is not included in our protocol, however we can include if the editor requires:  The researcher will **not** have access to these third party databases and will only obtain email addresses of potential participants if they choose to ‘opt-in’ to register to participate in the study. Furthermore, all contact with these participants will conform with the new GDPR regulations coming into effect in May 2018.  Once the stakeholder has received the invitation from whichever mailing list they have opted into receiving information from, they can freely decide whether they would like to participate or not. The invitation email will include an information sheet. This information sheet will explain the aims of the study, what participants are being asked to do and why their participation is important and contact information in case they require further information [See Appendix 2].  Registered participants who do not complete Round 1 will not receive Round 2 of the survey, likewise if participants do not complete Round 2 they will not be invited to complete Round 3. Participants who complete Round 2 and Round 3 will be invited to consider if they are willing to attend a face to face consensus meeting to discuss the final set of outcomes. Participants who would like to consider attending the face to face meeting will be contacted by the researcher via the contact information they provide |
| Data management | 19 | Plans for data entry, coding, security, and storage, including any related processes to promote data quality (eg, double data entry; range checks for data values). are as stated in our ethics application (as highlighted below), this text is not included in our protocol, however we can include if the editor requires:  Our data management plan has been set out in our ethical approval application. In Phase 2:All documents bearing personal information will be stored in a locked cabinet with access strictly restricted to the researchers on this study. All computerised data/information will be stored in a locked cabinet, again with restricted access and electronic material password protected.  Phase 3:  As there is a face to face component to this research the identities of the participants will be known to the research team. The participants will sign a written consent form identifying that they understand what the study involves and asking their permission to be photographed at the meeting. Consent forms will be stored in a locked cabinet with access strictly restricted to the researchers on this study. All computerised data/information will be stored in a locked cabinet, again with restricted access and electronic material password protected. |
| Statistical methods | 20a | Statistical methods for analysing primary and secondary outcomes. Reference to where other details of the statistical analysis plan can be found, if not in the protocol |
|  | 20b | NA |
|  | 20c | NA |
| **Methods: Monitoring** | | |
| Data monitoring | 21a | NA |
|  | 21b | NA |
| Harms | 22 | NA |
| Auditing | 23 | NA |
| Ethics and dissemination | | |
| Research ethics approval | 24 | Ethic approval has been granted from NUI Galway ethics application. Pg11 |
| Protocol amendments | 25 | Plans for communicating important protocol modifications (eg, changes to eligibility criteria, outcomes, analyses) to relevant parties (eg, investigators, REC/IRBs, trial participants, trial registries, journals, regulators) |
| Consent or assent | 26a | Pg 12 |
|  | 26b | Additional consent provisions for collection and use of participant data and biological specimens in ancillary studies, if applicable |
| Confidentiality | 27 | How personal information about potential and enrolled participants will be collected, shared, and maintained in order to protect confidentiality before, during, and after the trial |
| Declaration of interests | 28 | Financial and other competing interests for principal investigators for the overall trial and each study site |
| Access to data | 29 | Statement of who will have access to the final trial dataset, and disclosure of contractual agreements that limit such access for investigators |
| Ancillary and post-trial care | 30 | NA |
| Dissemination policy | 31a | Pg12 |
|  | 31b | Pg12 |
|  | 31c | Pg 12 |
| Appendices |  |  |
| Informed consent materials | 32 | NA |
| Biological specimens | 33 | NA |

*It is strongly recommended that this checklist be read in conjunction with the SPIRIT 2013 Explanation & Elaboration for important clarification on the items. Amendments to the protocol should be tracked and dated. The SPIRIT checklist is copyrighted by the SPIRIT Group under the Creative Commons “[Attribution-NonCommercial-NoDerivs 3.0 Unported](http://www.creativecommons.org/licenses/by-nc-nd/3.0/)” license.
