# Supplementary material for: COSUTI: a protocol for the development of a core outcome set (COS) for interventions for the treatment of uncomplicated urinary tract infection (UTI) in adults
Source: Trials. 2019 Feb 7;20:106. doi: 10.1186/s13063-019-3194-x (PMC6367821; doi:10.1186/s13063-019-3194-x)
Supplement: Supplementary file 2 — PubMed search queries for the systematic review. (DOCX 17 kb) [file 13063_2019_3194_MOESM2_ESM.docx]

### Additional file 2

### Pub Med Search Queries for Systematic Review

| Search | Query | Items found |
| --- | --- | --- |
| #1 | Search Urinary Tract Infection | 64252 |
| #2 | Search UTI | 8136 |
| #3 | Search cystitis | 13236 |
| #4 | Search (#1 OR #2 OR #3) | 75637 |
| #5 | Search Randomized controlled trial [pt] | 461856 |
| #6 | Search controlled clinical trial [pt] | 549442 |
| #7 | Search randomized [tiab] | 444871 |
| #8 | Search Placebo [tiab] | 194306 |
| #9 | Search Clinical trials as topic [mesh:noexp] | 183728 |
| #10 | Search randomly [tiab] | 291692 |
| #11 | Search trial [ti] | 182518 |
| #12 | Search (#5 OR #6 OR #7 OR #8 OR #9 OR #10 OR #11) | 1160741 |
| #13 | Search (animals [mh] NOT humans [mh]) | 4461191 |
| #14 | Search (#12 NOT #13) | 1069186 |
| #15 | Search (treatment* OR therap* OR technolog* OR strateg* OR arm OR intervention* OR method*) | 12570175 |
| #16 | Search (#4 AND #14 AND #15) | 5325 |
| #17 | Search (#4 AND #14 AND #15) Filters: Publication date from 2007/01/01 to 2017/12/31 | 1954 |

### Central Search Queries for Systematic Review

| Recent queries in Central |  |  |
| --- | --- | --- |
| Search | Query | Items found |
| #1 | urinary tract infection | 6650 |
| #2 | UTI | 1339 |
| #3 | Cystitis | 1334 |
| #4 | #1 or #2 or #3 | 8041 |
| #5 | Randomized controlled trial:pt | 453339 |
| #6 | Controlled clinical trial:pt | 430631 |
| #7 | randomised:ti,ab | 99428 |
| #8 | Placebo:ti,ab | 213401 |
| #9 | MeSH descriptor: [Clinical Trials as Topic] this term only | 34654 |
| #10 | randomly:ti,ab | 180300 |
| #11 | trial;ti | 1970 |
| #12 | #5 or #6 or #7 or #8 or #9 or #10 or #11 | 743999 |

### Embase Search Queries for Systematic Review

| Recent queries in EMBASE |  |  |
| --- | --- | --- |
| Search | Query | Items found |
| #1 | ‘crossover procedure’:de OR ‘double-blind procedure’:de OR ‘randomized controlled trial’: de OR ‘single-blind procedure’:de OR random*:de,ab,ti OR factorial*:de,ab,ti OR crossover*:de,ab,ti OR ((cross NEXT/1over*):de,ab,ti) OR placebo*de,ab,ti OR ((doubl* Near/1 blind*):de,ab,ti) OR ((singl*Near/1 blind*):de,ab,ti) OR assign*: de,ab,ti OR allocate*:de,ab,ti OR Volunteer*: de,ab,ti | 2,242,418 |
| #2 | ‘urinary tract infection’:ti,ab OR ‘uti’ OR cystitis’:ti,ab | 50,613 |
| #3 | Treatment* OR therap* Or technolog* OR strateg* OR intervention* Or method* | 17,88,315 |
| #4 | #1 AND #2 AND #3 | 5469 |
| #5 | #1 AND #2 AND #3 AND [English]/lim AND [2007-2017]/py | 3550 |
